# Pain Mitigation Strategies for Disbudding in Goat Kids

**DOI:** 10.3390/ani14040555

**Published:** 2024-02-07

**Authors:** Preet Singh, Dinakaran Venkatachalam, Kavitha Kongara, Paul Chambers

**Affiliations:** Tāwharau Ora School of Veterinary Science, Massey University, Palmerston North 4410, New Zealandk.kongara@massey.ac.nz (K.K.); j.p.chambers@massey.ac.nz (P.C.)

**Keywords:** disbudding, goat kids, analgesia

## Abstract

**Simple Summary:**

The process of removing horn buds (disbudding) is painful for young animals. People are increasingly concerned about the well-being of animals, so finding ways to reduce the pain and stress caused by disbudding is important. This review discusses various methods to ease pain during disbudding in goat kids, including using drugs to sedate and relieve pain, blocking nerves, and giving anti-inflammatory medications afterward. It also mentions the potential harm of certain drugs. The recommended approach is to use a combination of sedation, nerve blocking, and anti-inflammatory drugs for the best results in reducing pain. This review ends by suggesting directions for more research to further improve the well-being of young goats during the disbudding process.

**Abstract:**

Pain mitigation strategies for disbudding in goat kids have gained significant attention in recent years because of growing concerns for animal welfare. Disbudding, the removal of horn buds in young goats, is a common practice to enhance safety and manage herd dynamics. However, the procedure will cause pain and distress if not managed effectively. This review covers the array of pain mitigation techniques currently available for disbudding, including the efficacy of these strategies in reducing pain and stress during the disbudding process, with specific attention to the potential toxicity associated with local anesthetics. The current best practice for disbudding on the farm suggests sedation/analgesia with an alpha-2 agonist, the placement of a two-point cornual nerve block, and then an NSAID for postoperative pain. In conclusion, this review offers recommendations for future research directions aimed at enhancing the welfare of young goats subjected to the disbudding procedure. These suggestions hold the promise of fostering significant improvements in the overall well-being of these animals.

## 1. Introduction

Goat kids, particularly females from milking herds, are commonly subjected to disbudding to reduce potential injury to animals and humans from horns or entanglement in equipment in the milking shed. Goat kids are typically disbudded within the first week of life, most commonly via thermal cauterization using a hot iron. Hot iron disbudding is a painful procedure that requires pain relief to be provided to the animals undergoing this procedure to avoid a negative impact on their welfare. Hot iron disbudding is considered a significant surgical process and should be performed by a veterinarian or under their supervision. This is particularly important in goat kids because there is a significant risk of thermal brain damage and death. 

The need for disbudding can be circumvented by selectively breeding for polledness. Although this approach is effective in cattle, it tends to trigger severe reproductive complications in certain dairy goats with European lineage, such as the Saanen, Alpine, and Toggenburg breeds [1]. In these breeds, the existence of horns is governed by a recessive gene that leads to infertility. Consequently, female goats with a homozygous polled genotype will mature as infertile intersex individuals, while male goats with the same genotype face a heightened likelihood of developing sperm granulomas. Therefore, selective breeding for polledness is not beneficial for these goat breeds, and disbudding remains the only tool to eliminate the horns [1].

The pain from hot iron disbudding has at least two phases, which probably require different approaches to alleviation. The application of a hot iron causes intense acute pain, and then, the release of inflammatory mediators from the burnt tissue will cause a lower-grade but longer-lasting pain. Both phases should be treated to ensure animal welfare. There are a variety of ways of accomplishing this: general anesthetics and analgesics with or without local anesthetics and then non-steroidal anti-inflammatory drugs (NSAIDs) for postoperative pain. The efficacy of local anesthetics and general anesthetics for disbudding pain in goat kids has not been extensively investigated or reported in the literature compared with calves. Most of the drugs recommended or referenced in this review are used off-label, as they are not registered for use in food-producing animals. Additionally, the registration status and availability of these drugs differ across countries and regions.

## 2. General Anesthesia and Systemic Analgesia

Xylazine hydrochloride is probably the most commonly used alpha-2 adrenergic agonist in ruminant veterinary practice despite its association with significant complications like hypoxemia and pulmonary edema. These complications are linked to the activation of pulmonary intravascular macrophages [2] and may involve the release of TNF-Alpha. TNF suppression agents, such as choline chloride given before xylazine, have a small beneficial effect [3]. Other inflammatory mediators are almost certainly involved as well. In adult sheep, the degree of hypoxemia is similar to xylazine, romifidine, detomidine, and medetomidine [4]. Goats exhibit higher sensitivity to xylazine compared with other ruminants [5]. As a result, careful dose calculation and monitoring of the effects of xylazine are particularly important in goats, especially in goat kids, when compared with other ruminants.

Sedatives such as xylazine have proven effective in reducing stress during restraint and offer partial analgesic effects, yet they do not provide comprehensive pain relief during disbudding. Wagmann et al. (2018) investigated the efficacy of a mixture of xylazine (0.05 mg/kg) and ketamine (20 mg/kg) administered prior to disbudding by certified Swiss farmers [6]. The authors concluded that this mixture did not provide adequate anesthesia and analgesia in goat kids and suggested that refinement to this protocol is required. Dexmedetomidine administered intramuscularly may be more effective for analgesia as compared with the administration of lidocaine around the horn bud and intramuscular injections of meloxicam [7]. In the UK, where disbudding is carried out under general anesthesia in the first week of life, xylazine overdose is considered the most common cause of death [8]. Alphaxalone has been recommended for disbudding performed on the farm at a dose of 6 mg/kg administered intravenously [9]. As it provides negligible analgesia [10], a pre- and postsurgical analgesic protocol should be followed.

Alpha-2 agonists are easy to administer while performing this procedure on the farm; however, they produce adverse effects such as hypothermia and cardiovascular and respiratory depression and often require a reversal agent such as yohimbine or atipamezole postoperatively (which will also reverse analgesia).

Nonsteroidal anti-inflammatory drugs have demonstrated their ability to alleviate post-disbudding pain; however, they have fallen short in preventing the acute pain induced during the disbudding process itself [11]. Another disadvantage is that, as goats are disbudded shortly after birth, drugs requiring liver metabolism may have a variable, but probably long, duration of action. Goat kids, like other neonates, almost certainly have a restricted capacity to metabolize and eliminate drugs. The administration of sedatives such as xylazine may reduce stress and make it easier to administer local anesthetics.

Inhalant general anesthetics, especially isoflurane, have demonstrated effectiveness in mitigating pain during disbudding. However, their practicality in commercial farming scenarios is questionable given the need for anesthetic equipment and the potential for increased disbudding costs [12]. Using inhalation anesthetics in oxygen delivered by an anesthetic machine via a face mask used to be considered the best practice [9] but can be dangerous in the presence of a hot iron, as oxygen supports combustion.

The United Kingdom and several European countries mandate that disbudding in goats is exclusively carried out by a veterinarian, utilizing appropriate anesthetics and analgesics [6]. Consequently, in New Zealand and Australia, a significant shift has occurred, and pain relief measures have become mandatory for disbudding goat kids [13]. This represents a noteworthy advancement in animal welfare practices.

## 3. Local Anesthesia

**Figure 1:**
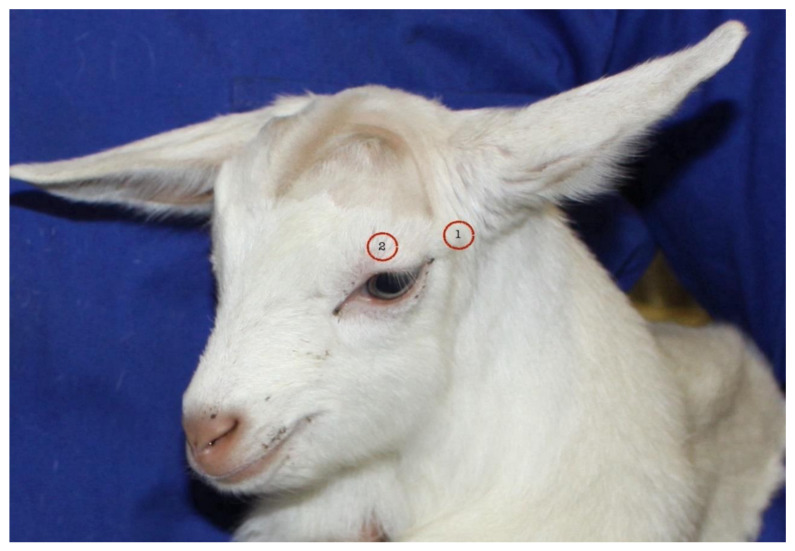
Each horn bud in the goat kids is innervated by two cornual nerves, one from the zygomaticotemporal nerve and the other from the infratrochlear nerve. Therefore, two injection sites (as shown in the image) on each side are recommended to effectively alleviate pain during the process of disbudding.

The administration of a local anesthetic to produce a nerve block is a common strategy for alleviating pain during the disbudding process [1]. Usually, two nerves are blocked on each side: the cornual branches of the infratrochlear nerve (a branch of the ophthalmic division of the trigeminal nerve CNV_1_) and the zygomaticotemporal nerve (also referred to as the cornual branch of the lacrimal nerve, a branch of the maxillary division of the trigeminal nerve CNV_2_) (Figure 1).

Usually, 0.5 mL of a local anesthetic is injected at each site, but because there may be several branches in the infratrochlear nerve [14], sometimes larger volumes are used in order to achieve a greater spread.

All local anesthetics have a potential for toxicity [15]. Goat kids, given their small size, are susceptible to overdosing. Overdose can cause cardiac effects (signs of reduced cardiac output), sedation, convulsions, and death. The need to block four nerves for both horn buds (in contrast to a single nerve per side in calves) increases the amount of local anesthetic required. Additionally, the vascularity at the site of the nerve block [16] exacerbates the risk.

Goat kids are not small calves [17], as they undergo disbudding at a younger age, with thinner skulls and much lower body weights [17]. For instance, goat breeds like pygmy and Nigerian Dwarf, which can weigh under 2 kg, might encounter issues when 2 mL of 2% lidocaine (20 mg/mL), equivalent to 20 mg/kg, is injected for nerve blocks. This dosage could easily lead to plasma concentrations sufficient to cause convulsions [18].

It is crucial to emphasize that the techniques used for disbudding calves should not be directly applied to goat kids. Thinner skulls in goat kids increase the risk of brain damage due to thermal injuries, resulting in convulsions and death. Pathological findings from goat kids disbudded with hot irons have revealed central areas of cavitation in the brain both in gray and white matter. Histological lesions included extensive hemorrhages and coagulation necrosis [19].

The thermal lesions caused by cautery disbudding can be infected by bacteria, leading to a potential risk of bacterial invasion and the development of meningoencephalitis [20]. This can be treated with a course of broad-spectrum antibiotics [19]. Surviving goat kids display signs of incoordination, paraplegia, and convulsions even up to 3 weeks. The application time is also crucial, as the placement of a cautery iron for 15 to 20 s has been shown to cause severe brain injuries [21].

## 4. Lidocaine

Lidocaine is the most commonly used local anesthetic in veterinary practice [5] in most places, apart from the EU, and is thus used in goat medicine. It is a cheap, effective, usually safe, and readily available drug. Only one study has reported convulsions in a goat kid following the intramuscular injection of lidocaine at approximately 10 mg/kg [22]. In a dose-ranging investigation, a dosage of 7 mg/kg of body weight administered intravenously over a 60 s interval yielded no observable signs of toxicity [18]. Consequently, this dosage is presumed safe for cornual nerve blocks in goat kids, apart from possibly accidental intravenous administration. This dose may also be deemed safe for other localized and regional nerve blocks in goat kids, although more extensive safety studies involving more animals are necessary.

The minimum dose necessary to induce seizures in young goats (12.42 mg/kg) [18] is lower than the comparable dose observed in newborn lambs (18.40 mg/kg) [22], and the average plasma level associated with convulsions in young goats (13.59 ± 2.34 µg/mL) [18] is lower than the level observed in newborn lambs (16.6 ± 1.2 µg/mL) [22]. Comparatively, in dogs, the lidocaine concentration leading to toxicity averages 8.21 + 1.69 µg/mL, which is notably below the level recorded in young goats (13.59 ± 2.34 µg/mL) [23]. Meyer et al. (2001) found that horses exhibited intoxication at a serum concentration of 3.24 ± 0.74 µg/mL [24]. These variations might stem from the diverse criteria used to assess toxicity across different species. In dogs, the toxic indicator is the tonic extension phase, and horses are evaluated based on skeletal muscle fasciculation [23,24], while for goat kids, the endpoint is the occurrence of convulsions [18].

An additional factor contributing to these observed disparities might be the variation in the rate of drug administration across different species or the fact that young animals display reduced sensitivity to lidocaine toxicity when compared with adult animals, a phenomenon likely attributable to the higher volume of distribution, a characteristic of younger individuals [22].

The subcutaneous administration of 0.5 mL of 1% lidocaine (such as around a superficial nerve) exhibits rapid absorption in goat kids, with an average T_max_ of 0.33 ± 0.11 h [18]. Rapid absorption has also been reported following cornual nerve blocks in goat kids [25]. The C_max_ (0.58 ± 0.17 µg mL^−1^) was around four times less than the concentration (2.55 ± 0.41 µg mL^−1^) observed at 1 min following intravenous administration of 8 mg kg^−1^ for over 60 s, the maximum dose that did not show any observable toxicity signs. The elimination rate of both lidocaine and its main metabolite in most species, monoethylglycinexylidide (MEGX), is moderate, indicated by mean t_1/2λz_ values of 2.28 h and 3.20 h, respectively. After subcutaneous administration, the mean peak plasma concentration of lidocaine (2.12 ± 0.81 µg/mL) is roughly 6.5 times lower than the mean plasma concentration associated with convulsions (13.59 ± 2.34 µg/mL) [11]. Decreasing peak plasma concentrations lowers the likelihood of encountering toxicity [26]. Given that the C_max_ resulting from a 0.5 mL/site injection of 1% lidocaine hydrochloride is significantly below the toxic plasma concentration, this dosage is likely to be safe for cornual nerve blocking in goat kids. No pain-related behavioral signs have been observed during disbudding, indicating that it is also effective. Nevertheless, most of the goat kids begin exhibiting behaviors like head scratching and head shaking around 20 min after nerve blocks, indicating that the anesthetic effect lasts only for about 20 min.

Another major concern of using lidocaine in food-producing animals is its metabolism to dimethylaniline, (DMA, 2,6 xylidine). Amide-type local anesthetics like lidocaine undergo hepatic biotransformation through specific cytochrome P450 isoforms (CYP3A4) [27]. In the liver, lidocaine is transformed into various metabolites, including MEGX and glycinexylidide (GX), via oxidative N-dealkylation and DMA via hydrolysis [28]. MEGX further undergoes biotransformation into DMA, although MEGX does not appear to be produced in significant quantities in adult cattle [29]. DMA is oxidized in the liver into N-(2,6-dimethylphenyl) hydroxylamine (DMHA) and 4-amino-3,5-dimethylphenol (DMAP). DMHA then goes through phase II biotransformation, specifically acetylation, forming reactive esters that ultimately convert into a reactive nitrenium ion through phase 2 metabolism [27]. Both DMHA and the nitrenium ion can bind covalently to DNA, potentially leading to the development of tumors. DMHA can also react with hemoglobin, resulting in the formation of hemoglobin adducts through covalent binding with cysteine residues [27]. The other metabolite, DMAP, undergoes oxidation into an iminoquinone, a highly reactive electrophile with genotoxic properties [30]. DMAP can also be generated from the nitrenium ion or DMHA. DMA is also rapidly converted into 4-hydroxyDMA, which accounts for most urinary excretions in adult cattle [29]. DMA, based on toxicology studies in rats, has been categorized as a possible carcinogen (Group B) by the International Agency for Research on Cancer [31]. Their study revealed that the chronic oral administration of DMA at a dosage of 3000 mg/kg resulted in the development of various cancers in rats, including nasal papilloma and carcinoma, rhabdomyosarcoma, subcutaneous fibromas, and fibrosarcomas [32]. Furthermore, the mortality rates were higher in animals that received 1000 and 3000 mg/kg of DMA compared with a control group. These findings led to the conclusion that DMA acts as a carcinogen in rats. In dogs, chronic oral administration of DMA causes weight reduction, hyperbilirubinemia, hypoproteinemia, and significant fatty degeneration. In vitro studies have further confirmed the genotoxic and mutagenic properties of DMA [27]. Several human studies have also indicated a potential link between DMA and an increased risk of bladder cancer [33,34]. Collectively, these reports strongly suggest that DMA may indeed be a carcinogen (at high doses), making the use of lidocaine in animals undesirable.

## 5. Articaine

Articaine is also an amino-amide class of local anesthetic and has a distinct molecular structure with a thiophene ring instead of a benzene ring, as well as an ester group. The presence of the thiophene ring enhances its lipid solubility, enabling articaine to penetrate nerve fibers more efficiently and rapidly [35]. Moreover, its unique molecular composition allows for improved penetration through both bone and soft tissues compared with other local anesthetics [36,37]. The ester group present in articaine makes it noteworthy among amino-amide local anesthetics, as it undergoes rapid hydrolysis via esterases in tissues and plasma [35,36,37] into inactive articainic acid. Its structure means that DMA is not formed during metabolism. Therefore, articaine hydrochloride could be a safer and better option than lidocaine in goat kids.

An investigation involving goat kids was conducted to assess the toxicity and pharmacokinetics of articaine [25]. This study involved the intravenous administration of articaine hydrochloride IV over a 60 s interval, and cornual nerve blocks were administered using 1.5% articaine hydrochloride at a dosage of 0.5 mL per site. The goat kids exhibited no indications of toxicity associated with nerve blocks throughout these procedures. The average IV dosage required to produce convulsions was 16.24 ± 1.79 mg kg^−1^, and the average plasma concentrations of articaine and articainic acid at convulsions were 9.90 ± 2.38 µg mL^−1^ and 1.52 ± 0.91 µg mL^−1^, respectively.

A cornual nerve block (0.5 mL/site) using 1.5% articaine hydrochloride took about 4 min to anesthetize the horn buds based on the absence of a withdrawal response and vocalization during disbudding. This confirms the effective analgesic properties of articaine. However, post-procedure observations of the animals indicated the emergence of pain-related behaviors, including head scratching and head shaking, approximately 25 min after administration [25].

The absorption of articaine after cornual nerve block is rapid with a mean C_max_ of 586.58 ± 175.10 ng mL^−1^ at 0.22 ± 0.09 h (T_max_) [25]. The rapid absorption of articaine has also been reported in red deer [38] and humans [39]. This could be because of vasodilatation, similar to most other local anesthetics [40,41]. The short elimination half-lives (t_½λz_) of articaine following intravenous administration (0.66 ± 0.14 h) and subcutaneous administration (1.26 ± 0.34 h) indicate that articaine is rapidly eliminated following systemic absorption [25]. Rapid elimination has also been reported in deer [38] and people [39]. The rapid elimination of articaine may be due to rapid hydrolysis via plasma esterases into articainic acid [37]. Articainic acid is an inactive metabolite, whereas several primary metabolites of lidocaine are active and can increase the risk of toxicity during accidental intravenous administration or overdosage [35,42]. The plasma clearance of articaine was rapid in goat kids with a mean CLss of 5.33 ± 0.66 L kg^−1^ in [25].

There have been no documented cases of toxicity associated with articaine in humans, except, rarely, paresthesia. Paresthesia is characterized by persistent anesthesia or altered sensations in the form of neuropathy [43]. The underlying causes of paresthesia are still not fully understood, although it is believed to be related to the concentration of local anesthetics used. It has been observed that paresthesia occurs more frequently following the administration of 4% local anesthetic formulations, such as articaine and prilocaine, compared with the use of 2% local anesthetic formulations [43] (Puccini et al., 2015).

While articaine is generally regarded as a safe local anesthetic, it can potentially cause systemic toxicity (including central nervous system and cardiovascular toxicity) in the same way as other local anesthetics when toxic concentrations are achieved through inadvertent intravenous administration or overdose. However, the risk of systemic toxicity from overdosing is relatively low compared with lidocaine and other amide-type local anesthetics, as articaine undergoes rapid hydrolysis following systemic absorption [35]. The clearance of articaine was found to be 10 times greater than that of lidocaine in humans [44]. Rapid hydrolysis into an inactive metabolite and rapid elimination indicate that articaine may be safer than lidocaine for cornual nerve blocks in goat kids. Because of its wider margin of safety, articaine is clinically used as a 4% solution, whereas lidocaine is used as a 2% solution [37].

## 6. Bupivacaine

Both lidocaine and articaine are effective for less than 30 min. The most commonly used local anesthetic in people, bupivacaine, has a much longer duration in most species. It was used for nerve blocks in adult goats in [45] for the surgical examination of the stifle joint conducted under general anesthesia and sciatic–femoral nerve blocks. In that study, 0.5% bupivacaine provided effective analgesia with minimal adverse effects. A group receiving a higher dose of bupivacaine exhibited unilateral motor blockade. There are no reports of its use in goat kids. Extended-release formulations of bupivacaine have been used for disbudding in calves [46]. However, long-acting local anesthetics such as bupivacaine may not be a good choice for goat kids, as bupivacaine possesses a greater risk of cardiac toxicity than articaine and lidocaine. This is an area that needs further research.

## 7. Postoperative Analgesia

Since many of the currently available local anesthetics for farm animals are short-acting, additional pain relief, such as systemic NSAIDs, may be required to control post-disbudding pain. Meloxicam has been reported to reduce post-disbudding pain in goat kids [12]. The subcutaneous administration of meloxicam one hour prior has been shown to minimize the expression of inflammatory cytokines in calves [47]. The administration of both local anesthesia and systemic NSAIDs appears to have the potential to provide better analgesia (compared with their single use) for disbudding pain. However, this did not provide complete postoperative pain relief in goat kids [11,48]. A mixture of meloxicam, lidocaine, and xylazine demonstrated greater effectiveness in the initial hour compared with using only lidocaine [48]. Therefore, a multimodal analgesic approach is likely to prove more beneficial than the use of a single class of drugs.

## 8. Alternative Methods of Disbudding

Various alternative methods to thermal cautery disbudding have been explored in goat kids [49]. These include cryosurgical and chemical disbudding techniques. Cryosurgery involves the use of liquid nitrogen, while the chemical method employs caustic paste (usually sodium, calcium, or potassium hydroxide paste) to destroy horn buds. However, both cryosurgical and caustic paste methods have been shown to induce more pain than cautery disbudding, as indicated by physiological and behavioral changes [49]. Caustic paste can also pose the risk of damaging the eyes of goat kids and the udders of does.

There is a need for a simple, safe, cost-effective technique to prevent the growth of horn buds in goat kids. An approach involving clove oil injection into the buds has been explored for horn bud destruction both in goat kids [50] and in calves [51], but the degree of distress experienced during or after the procedure has not been extensively reported. Histopathological changes in horn buds injected with clove oil have revealed coagulative necrosis of the epidermis and the infiltration of neutrophils. In a pilot study involving 12 calves injected with clove oil and isoeugenol at different volumes, it was found that the injection volume plays a crucial role in successful disbudding in calves [52]. Although clove oil or isoeugenol injections cause less tissue damage compared with hot iron disbudding, the success rate is lower, as scur formation was observed six months post-procedure [53,54]. Therefore, clove oil injection may not be entirely effective in preventing horn bud growth, as evidenced by the emergence of scurs in a substantial number of goat kids.

Cloves have been used as an anesthetic for fish [55] and a product (Aqui-S, Aqui-S New Zealand Ltd., Lower Hutt, New Zealand) is currently registered to enable handling. It has traditionally been used as a local anesthetic agent in human dentistry; however, at higher doses, it exhibits cytotoxic effects [56]. As cloves and their oil have been used since antiquity as food flavorings, there are fewer concerns about residues in food-producing animals. Consequently, these characteristics render them a viable alternative for disbudding.

Concerns regarding adverse effects in goat kids, including swelling around the horn buds, have been raised because of hypersensitivity reactions caused by eugenol, the main component of clove oil [49]. The pain experienced during the administration of clove oil for disbudding in calves has been found to be significantly less than the cautery disbudding method. However, calves injected with clove oil still exhibited signs of discomfort in [57].

A similar study with calves subjected to clove oil injection showed that, while it might delay horn bud growth, complete elimination is not always achieved. Over a 16-month observation period, calves developed horns or scurs, suggesting that clove oil injection might not offer full prevention of horn bud growth [58]. These findings emphasize the complexities and limitations associated with alternative disbudding methods in terms of both effectiveness and potential adverse reactions.

While clove oil appears promising as an alternative to cautery disbudding, it falls short of the effectiveness demonstrated by traditional methods. Further efforts should be directed toward refining the formulation of clove oil or eugenol to enhance its distribution in the horn bud. Research is essential to understanding the pharmacokinetics and systemic absorption of eugenol/clove oil and determining if a slow-release formulation is necessary. Such advancements could potentially reduce the volume used and mitigate associated side effects.

## 9. Conclusions

The ideal analgesia for goat kid disbudding is probably a combination of general anesthetic with postoperative analgesia, but this is not practical or economical on a farm. The current best practice for disbudding on the farm suggests sedation and analgesia with an alpha-2 agonist, the placement of a two-point nerve block, and then an NSAID for postoperative pain. Even this is unlikely to be completely effective in all cases, and more research is needed in this area.

Local anesthetics are an essential part of the analgesic protocol, but goat kids are susceptible to overdose. Articaine has been shown to be a safe and effective local anesthetic for cornual nerve block in goat kids with some potential advantages over lidocaine. However, comprehensive future studies, involving different doses and concentrations of articaine hydrochloride within a larger population, are essential to definitively establishing its safety and efficacy for disbudding. Articaine is not registered for use in livestock and lacks defined minimum residual limits (MRLs). This absence of MRLs is a significant obstacle hindering its use in livestock. Residue studies to allow an MRL to be set for articaine would require substantial financial investment and a collaborative effort from the animal industry.

In addition to local anesthetic nerve blockades, sedation with an alpha-2 agonist will minimize stress and pain during local anesthetic injections. As recovery may be prolonged in neonatal animals, an antagonist should be available. Postoperative pain management using NSAIDs is essential, although long-acting local anesthetics may be available in the future. Evaluating the safety and efficacy of this protocol and its administration via alternative routes, such as transdermal or oral, for disbudding in goat kids should be a focus of future studies.

Looking ahead, further research into drugs that will prevent the growth of horn buds safely and effectively is needed. Addressing the issue of pain associated with disbudding in goat kids necessitates a collaborative effort across multiple disciplines, including clinical veterinarians, pharmacologists, chemists, and animal welfare scientists. By pooling expertise from these diverse fields, a more comprehensive and effective solution may be developed to enhance the well-being of goat kids during the disbudding process. This multidisciplinary approach is crucial for the successful resolution of the problem and the development of humane practices in goat farming.

## Data Availability

Not applicable.
